# Fluence and Dose Distribution Modeling of an Ultraviolet Light Disinfection Process for Pathogen Inactivation Efficiency Evaluation

**DOI:** 10.1021/acsomega.4c05715

**Published:** 2025-01-30

**Authors:** Tamás Dóka, Péter Horák

**Affiliations:** Department of Machine and Product Design, Faculty of Mechanical Engineering, Budapest University of Technology and Economics, Budapest 1111, Hungary

## Abstract

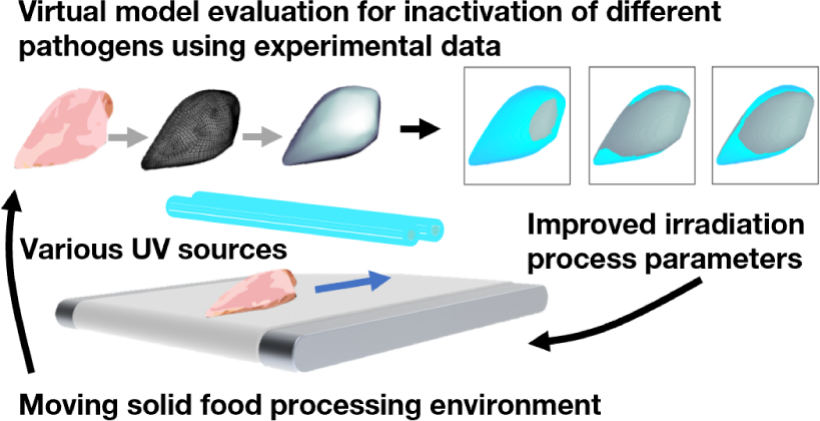

This study addresses the need to utilize bench-scale experimental results for ultraviolet (UV) light disinfection on solid food surfaces by proposing a novel framework to evaluate the fluence rate field of arbitrarily placed UV sources to ensure proper disinfection in industrial-scale food processing. Despite extensive research establishing UV fluence values for disinfection of various food types, industrial applications often face challenges due to nonhomogeneous UV distribution. This study introduces a method capable of determining the fluence distribution on solid food and food contact surfaces in both static and moving environments. Additionally, it aids in selecting the appropriate light sources and irradiation times. Our model leverages UV radiation models from different engineering disciplines to determine the UV fluence and dose distribution on the surface of convex objects. This helps to understand and optimize processes for proper decontamination, improved food quality, and a longer shelf life for processed products.

## 1 Introduction

The use of ultraviolet (UV) light emitters across diverse applications, leveraging their disinfectant capabilities in the food industry,^1−3^ medical applications,^4−6^ and water treatment sectors,^7^ as well as for inducing photochemical reactions in surface treatment,^8^ has become widespread. Precise models have been created and validated to simulate and evaluate the radiation field of various light emitter types for optimal utilization.^9,10^

The earliest radiation models were used to obtain proper dimensions for annular photoreactors, which are utilized for disinfection and other photochemical processes in liquids. In these applications, cylindrical, mercury-based UV light emitters are the focus of the models,^11^ restricting their general usability for different applications like surface treatment. The most precise models for mercury-based cylindrical light emitters are the superficial (extensive superficial diffuse emission model—ESDE) and volumetric (extensive source volumetric emission model—ESVE) models for near-field applications, and with significantly lower complexity, the line source diffuse emission model (LSDE) for far-field applications.^12^ For cylindrical (mercury free) excimer lamps, whose emission characteristics are fundamentally different from the mercury-based lamps, the complex SPACE (surface power apportionment for cylindrical excimer lamps) model was developed.^13^ With promising technological advancements, light-emitting diode (LED) UV emitters have become available, offering several advantages over conventional low-pressure mercury-vapor (LPM) UV lamps.^14^ However, LEDs have smaller output optical power and a higher relative price to date.^15^ Most LEDs can be modeled as point sources,^10^ enabling fast evaluation of their radiation field.

Typically, these models evaluate only the *fluence rate* field of the light emitters, which is used to determine the rate of disinfection processes in liquids or gases, where the direction of the received radiant flux is not relevant. In surface disinfection, these models must be adapted to account for the shape of the radiated object to properly evaluate the fluence rate distribution on the object’s surface. UV detectors measure the angle-dependent *irradiance* when assessing UV radiation. When surfaces are exposed to UV light during UV processes or when measuring UV doses with radiochromic films, the incidence angle of the light plays a considerable role. In well-designed bench-scale experiments, a homogeneous light distribution is created, where the fluence rate and irradiance are virtually the same.^16^ In general applications, this homogeneity cannot be ensured; therefore, along with the fluence rate, the irradiance distribution of UV sources needs to be evaluated to validate the simulation results or to calculate the received dose of the surfaces.

Bench-scale UV inactivation measurements have been conducted for various pathogens.^17^ Additionally, it has been demonstrated that different UV light source types, with varying emission spectra, can be more effective against some pathogens and less effective against others.^18^ However, due to differences in surface geometries and properties, pathogens require different amounts of fluence on different object surfaces. Bench-scale experiments have also investigated pathogen inactivation in solid food products^19,20^ and on different contact surfaces,^21^ enabling the creation of pathogen reduction models specific to these surfaces. Although shadowing usually does not occur on the macro-scale in bench-scale experiments, surface geometry at the microscale plays a significant role in the efficacy of the disinfection process. Surfaces with higher roughness are more susceptible to microscale shadowing, where pathogens can evade UV radiation.^21^ These inactivation models can be used to determine the UV process parameters at an industrial scale. However, careful evaluation of the fluence distribution on the irradiated object is essential to ensure the desired effect.^22^

This paper aims to translate bench-scale experimental results from existing literature into industrial applications by describing general models based on established and validated methods for fluence rate and irradiance calculations. Our model considers only direct UV radiation on the surface of a convex 3D object, where self-shadowing does not occur at any surface point on the macro-scale. However, for nonconvex objects or when multiple objects are evaluated simultaneously, shadowing can occur, significantly impacting the results. To model the shadowing effect, ray-tracing-based methods can be employed, although they require more computational power and time to accurately evaluate irradiance fields.^23^

In this study, radiation models that can be efficiently evaluated by computer programs are described for collimated light beams (directional sources), LEDs (point light sources), and LPM lamps (diffuse line sources). More complex models, such as the ESDE and VSDE for LPM sources, the SPACE model for cylindrical excimer lamps, or ray-tracing-based models to account for shadowing effects, are not considered. Using these models, we can calculate the combined fluence received from multiple arbitrarily placed light sources on a moving or static object, providing a foundation for process optimization.

Our method employs a virtual environment where light source setups are recreated to simulate the irradiation of a moving object. First, we present the inputs for environment creation and object motion model processing. Then, we describe the generalization of the fluence rate and irradiance calculation between a light source and a surface point, along with the radiation models of different light sources. After introducing pathogen inactivation models, we demonstrate evaluation of the fluence rate and irradiance fields. Finally, we discuss the potential use of our model for validation and optical output power measurement.

## 2 Materials and Methods

**Figure 1:**
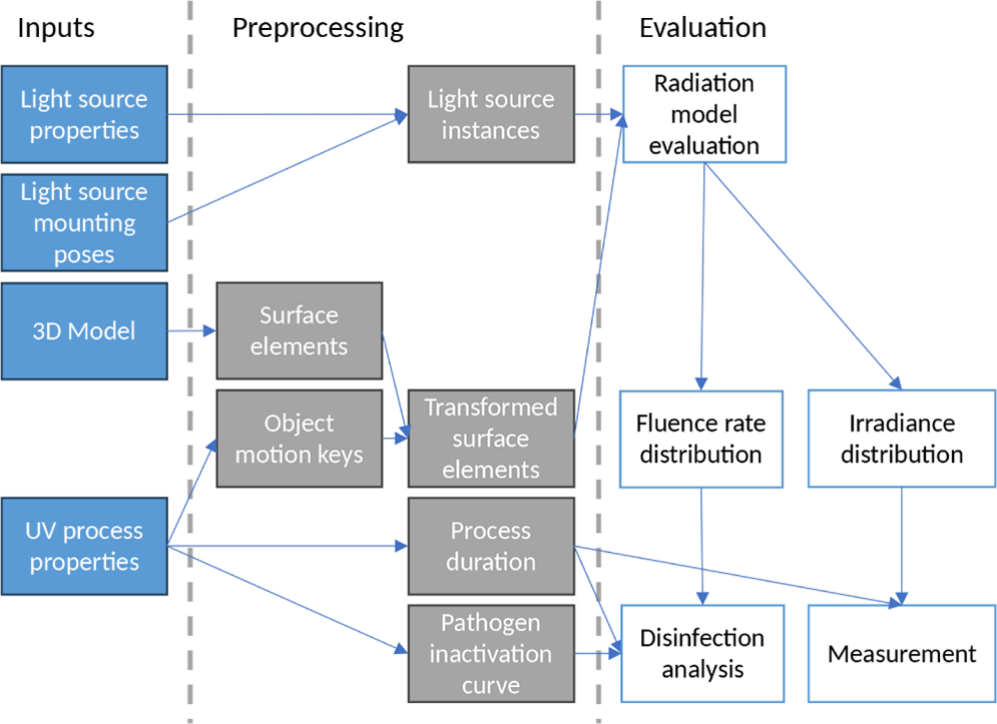
Evaluation process flowchart.

To evaluate the effectiveness of a UV process, the following information is needed: the type and mounting positions of the UV sources, the shape and size of the radiated object (3D model), the description of the object motion, the duration of the radiation, and the pathogen inactivation characteristics on the radiated object. Figure 1 shows the layout of the evaluation process, which consists of preprocessing of the inputs and evaluation. The main building blocks of the process are presented in the following sections.

### 2.1 Preprocessing

#### 2.1.1 Light Sources

Each light source used in the evaluation must be described by its mounting pose and radiation properties. The mounting pose (center position and orientation of the light source) can be represented in a homogeneous transformation matrix, relative to the origin of the base coordinate system. The required radiation properties vary for different types of light sources. For point sources (LEDs), the emission spectrum, emitted optical power or the maximum intensity value, and the relative intensity function are needed. For cylindrical sources (LPMs), the emission spectrum, emitted optical power, and radiating length are required.

For point light sources, estimation of the relative intensity function and the maximum intensity value are needed to obtain intensity values at any emission angle. This function can be approximated as a cosine function for nearly perfect cosine emitters.^8^ However, the measured relative intensity profile of a point source usually cannot be precisely estimated with the ideal source mentioned above for accurate results. Better models have been proposed in closed-form equations,^24,25^ but these models cannot be universally applied to all LED types.

**Figure 2:**
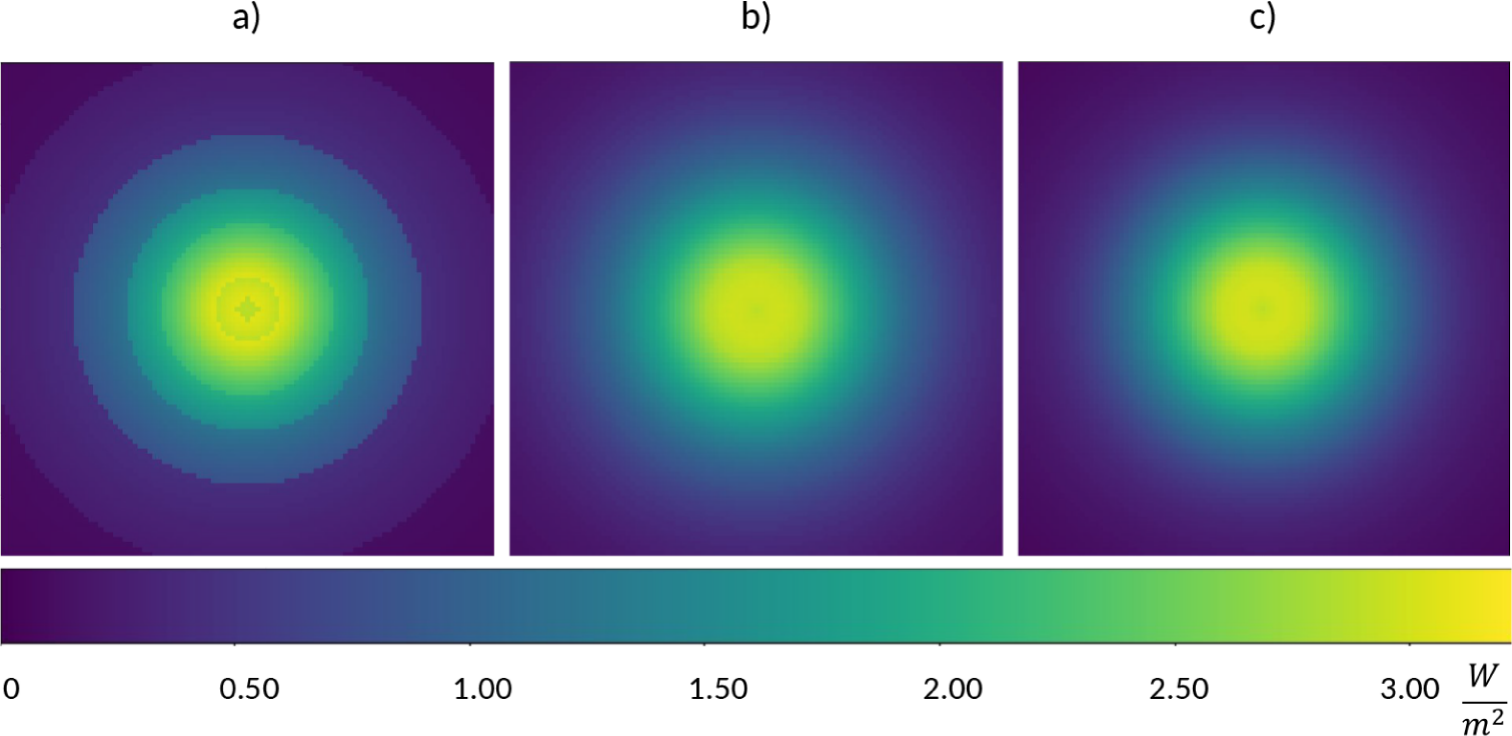
Difference between relative intensity estimations: a) closest value, b) linear interpolation, and c) FFT reconstruction—fluence rate values on a plane facing the LED light source (with 110 mW total radiated power) perpendicularly at 100 mm distance.

A straightforward approach is to create a lookup table from the manufacturer’s datasheet values and use the closest value for each input angle. For more precise results, linear interpolation between the datasheet points is a good alternative, or advanced numerical methods such as fast Fourier transform (FFT) can be used to reconstruct the intensity distribution. Figure 2 shows the results with different approaches using an LED emitter facing a plane.

In the examples, the Nichia NCSU434C (110 mW optical radiated power between 260 and 310 nm, 280 nm peak wavelength with approximately a 10 nm full width at half-maximum (fwhm)) UVC LED model was used for point light sources. The relative intensity function was estimated from the datasheet points (can be found in the Supporting Information) with linear interpolation. The maximum intensity was estimated to 0.036 [W/sr].

For the cylindrical LPM lamp model, properties of the Philips TUV PL-S 9*W*/2P 1CT (2.5 W optical radiated power (after 100 h) at 253.7 nm peak wavelength with a narrow emission band, fwhm under 5 nm) were used. The lamp has a compact twin-tube design, which was modeled as two parallel single-tube lamps positioned 14 mm apart with a radiating arc length of 129 mm. These compact models are shorter than conventional single-tube LPM lamps and are mounted on one end, allowing for more flexible designs. Therefore, this type of light source was selected for a comparison study.

#### 2.1.2 Environment

**Figure 3:**
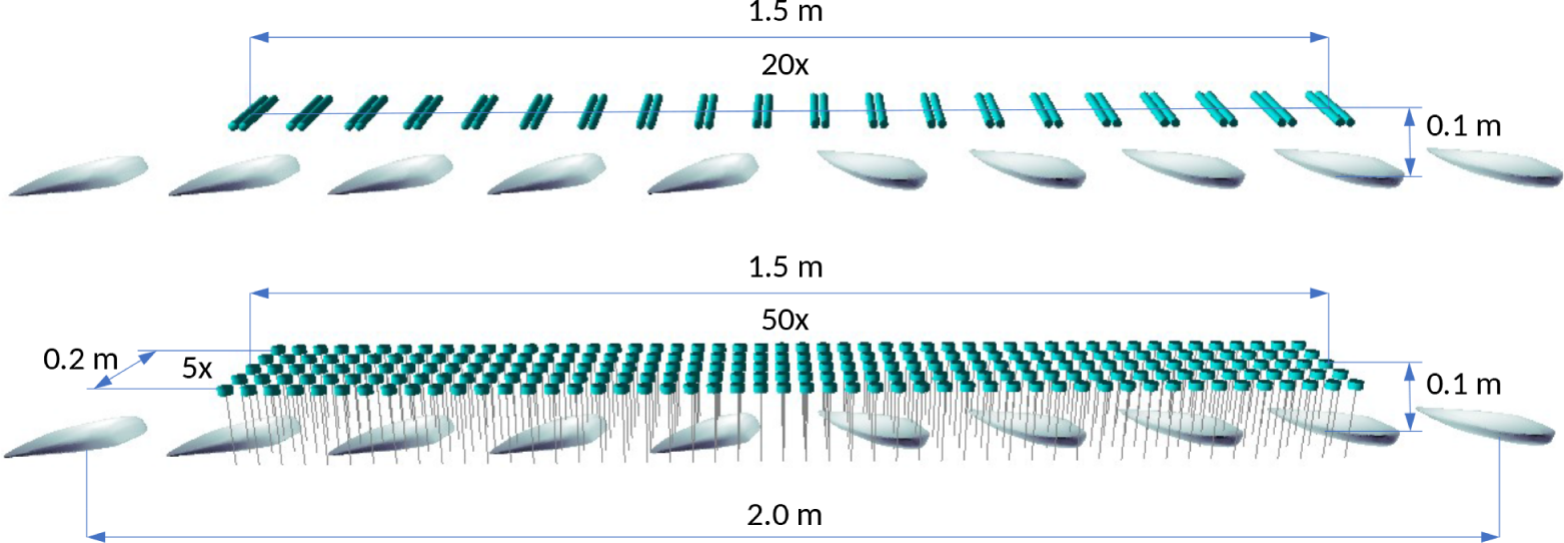
Light environment examples with similar layout and radiated power: 20 LPM light source (top) and 250 LED light source (bottom) at 0.1 m height above the base XY plane.

This study evaluates two virtual light source setups with similar outputs. Both are mounted on a plane parallel to the XY plane, 0.1 m above the origin. In the LPM setup, 20 Philips TUV PL-S 9*W*/2P 1CT lamps are evenly distributed along the *X*-axis over a 1.5 m length, with a central axis direction of [0, 1, 0] and a total combined radiated power of 25 W toward the base XY plane. The LED setup consists of 50 × 5 Nichia NCSU434C LEDs evenly distributed over 1.5 m along the *X*-axis and 0.2 m along the *Y*-axis. The central axis direction of the LED sources faces the base XY plane ([0, 0, −1]), and the total combined radiated power toward the base plane is 27.5 W. Figure 3 shows the layout of the virtual environments.

#### 2.1.3 3D Object Models

To efficiently evaluate the received fluence rate or irradiance on the surface of a convex 3D object, its surface points and their corresponding local surface normals are needed. First, a 3D model of the radiated object should be created on a 1:1 scale. Then, the convex hull of the model should be generated and stored in a quasi-uniform polygon mesh file (e.g., in an STL (*stereolithography*) file format). The convexity of the 3D object ensures that the object model does not cast a shadow on itself; hence, each surface point can be evaluated independently. Polygon meshes consist of triangles (or facets) and the corresponding surface normals, which serve as the input of our algorithm. During preprocessing, *surface elements* are extracted from the polygon mesh: the centroid of each mesh triangle is calculated to have a single surface point, and its surface normal to represent a small surface.

**Figure 4:**
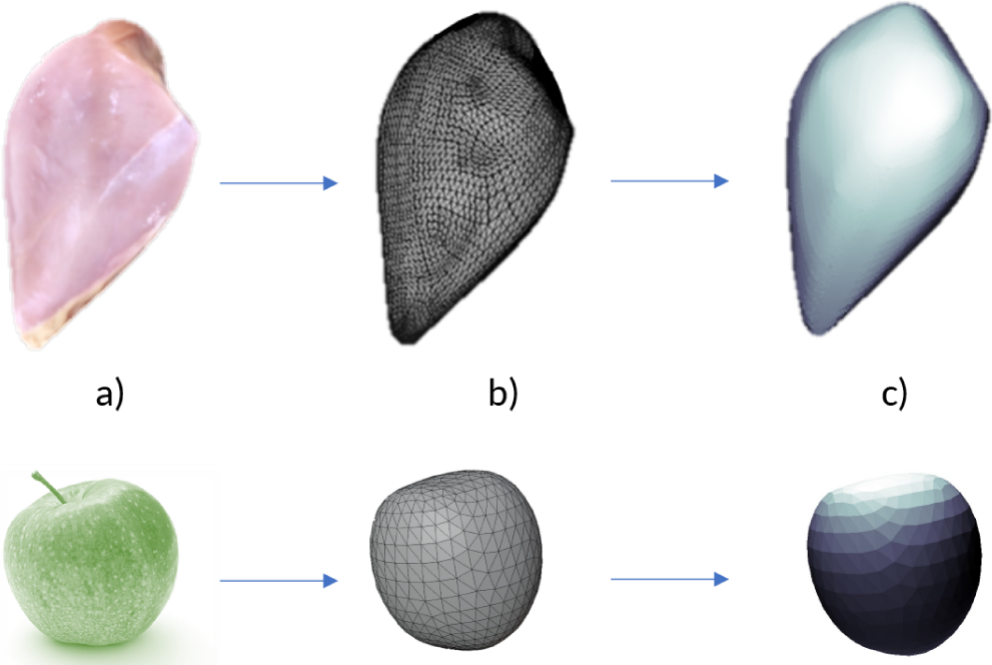
Example of processing 3D models (raw chicken breast (top, photo by PaShok3D from Sketchfab), apple (bottom, photo by mali maeder from Pexels)): a) real object, b) triangulated, quasi-uniform convex hull mesh, and c) evaluated radiation field on the surface of the object.

To demonstrate the evaluation process, a 3D model^a^ of a raw chicken breast (scaled to 165 mm length, convex hull generated, 43 212 triangles) and a 3D model of a simple apple (scaled to 60 mm diameter, convex hull generated, 1134 triangles) were used (Figure 4).

#### 2.1.4 Object Motion Modeling

**Figure 5:**
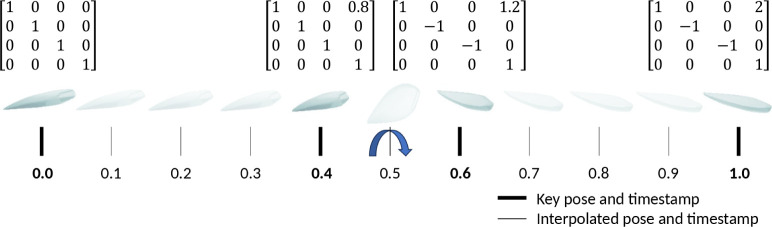
Example of object motion modeling, using key poses at normalized key timestamps. The object poses are written in a homogeneous transformation matrix form. Interpolated poses for 10 steps are also shown for linearly moving the object and flipping it in the middle of the motion.

The position and orientation of the radiated object and the light sources in the base coordinate system can be described by homogeneous transformations. The fluence rate distribution should be reevaluated at multiple positions when the object moves relative to the stationary light sources during radiation. The general motion of an object can be described with key poses at normalized key timestamps. Figure 5 shows the motion description of a raw chicken breast on a conveyor belt moving at a constant speed with a flip at the middle of the process, which was used for the evaluation. By setting the number of intermediate steps, first, timestamps are generated evenly; then, for each generated timestamp, the corresponding pose is interpolated, using linear interpolation for the intermediate positions and spherical linear interpolation (SLERP) for intermediate orientations from the SciPy python package.^26^

After the interpolated poses are obtained, the surface elements extracted from the object model are transformed with them, resulting in a set of transformed surface elements evenly distributed in time for each original surface element. Using the transformed surface elements, the received fluence rate can be evaluated for each surface element for each timestamp. By increasing the number of intermediate steps, the accuracy of the total received fluence can be improved, although at the expense of an increased runtime.

In the virtual LPM and LED setups, the object motion described above, with 50 steps and 1200 s duration, was evaluated.

### 2.2 Radiation Models of Light Sources

**Figure 6:**
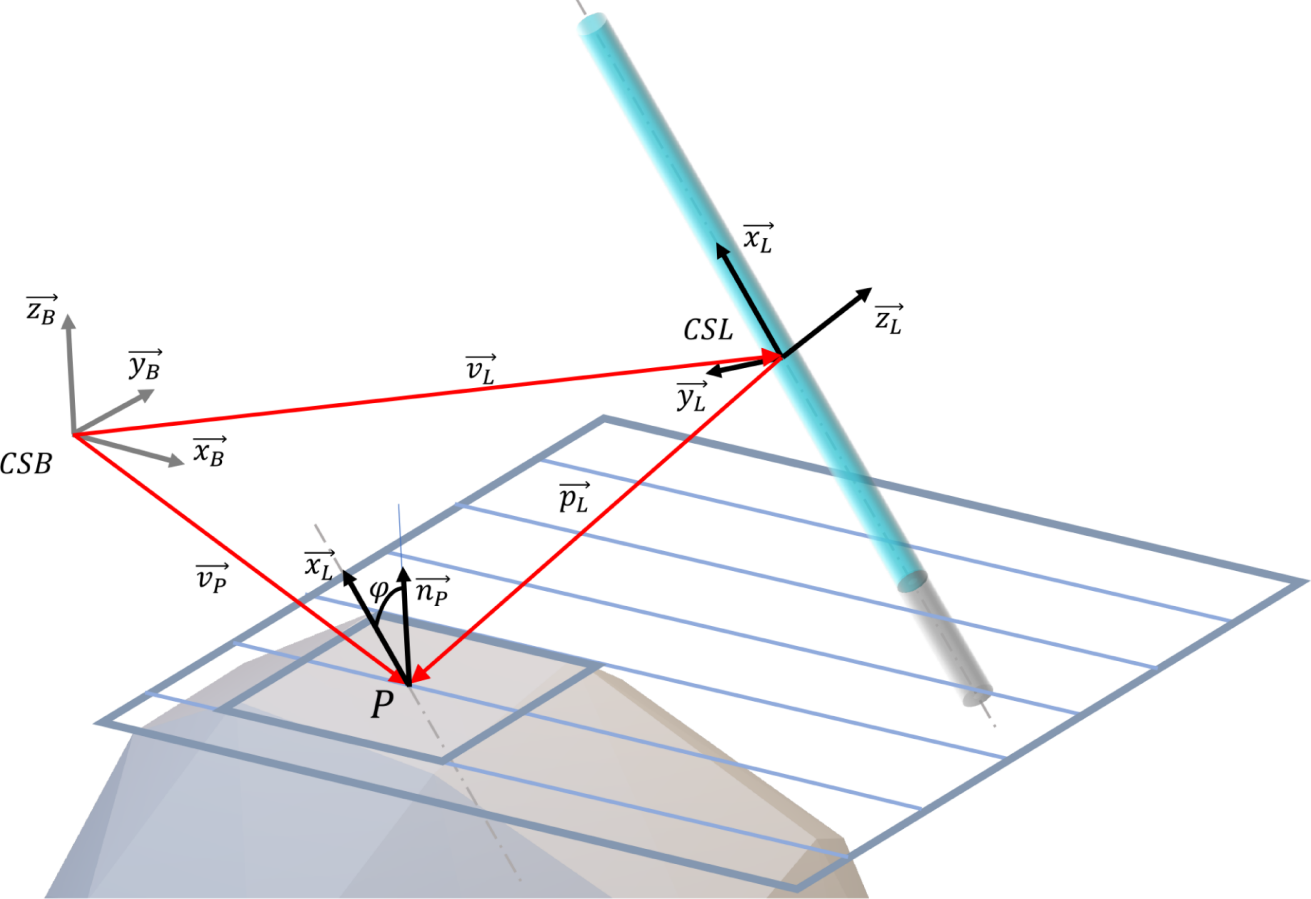
General arrangement of an object point *P* with position vector , local surface normal  and a line light source described with the position vector of its center point  and direction vector of its center line  inside the base coordinate system CSB.  gives the relative position of *P* from the lamp center point. In the lamp-focused approach, equations are described in local (lamp) coordinate system CSL.

Different models are needed for different kinds of light sources to correctly calculate the fluence rate and irradiance values on the surface of a convex object received from a light source. However, the general handling of these models should occur within the same base framework. In this framework, a radiated point of an object is described with its position vector () and local surface normal (), and a light source is described with its pose: center point position vector () and orientation (local coordinate system (CSL)); and its physical properties. The general arrangement of an object point and a light source is shown in Figure 6.

Evaluating the fluence rate and irradiance fields of the light source models in the following generalized closed forms allows for fast computation, making these models suitable for effectively assessing a large number of object points irradiated by multiple light sources. A detailed explanation of the different light source models used in this study to evaluate the fluence rate and irradiance distributions can be found in the Supporting Information.

#### 2.2.1 Directional Light Source Model

For directional light, like a collimated light beam, or solar light, the source can be described by its fluence rate , and direction vector of the light rays .

The fluence rate at the examined point is calculated as written in eq 1.
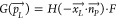
1

And the irradiance can be calculated as shown in eq 2.
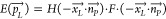
2

where  is the Heaviside step function, which is zero for negative numbers and constant one for non-negative numbers, to consider surface elements that are not facing the light source and, hence, are in shadow.

#### 2.2.2 Point Light Source Model

For point light sources, like most LEDs, the emitter can be described by its maximum intensity  (radiant intensity of the light source at the angle where the radiation is the strongest), angle-dependent relative intensity function  (which, when multiplied by *I*_max_ gives the intensity value at the angle measured from the optical axis), position vector of the lamp center  and direction vector of the lamp axis . Here,  represents the central axis of the point source.

The fluence rate and irradiance at the examined point can be described with eqs 3 and 4.
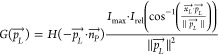
3
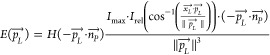
4

#### 2.2.3 Line Light Source Model

For cylindrical light sources, the line source models are the simplest yet still adequate with considerably low inaccuracies when the distance between the radiated point and the source is relatively high compared to the radius of the lamp.^27−29^ A lamp can be described by its total emitted UV optical power *P*_o_, radiating length *L*, position vector of the lamp center  and direction vector of the lamp axis .

In the LSDE model in fluence rate calculations, it is assumed that the examined point is radiated by the total length of the light source.^11^ However, in a general arrangement, when the local surface plane of the examined point intersects the lamp at its radiating length, radiation is received only from the segment of the lamp that extends above the examined point (the effective radiating segment). The effective radiating segment (*L*_+_, *L*_–_) can be calculated as shown in eqs 5,6.
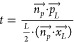
5
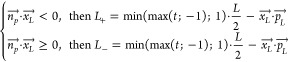
6

Knowing the limits of the effective radiating segment, the fluence rate and irradiance values can be obtained in a closed form for the diffuse radiation model (eqs 7,8):
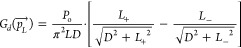
7
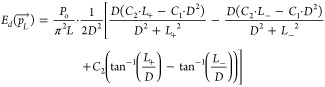
8

The constants *D*, *C*_1_ and *C*_2_ can be calculated for each examined point as seen in eqs 9–11.
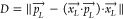
9
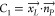
10
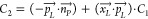
11

### 2.3 Evaluation of Fluence Rate and Irradiance

After the inputs were preprocessed, the effect of the light source instances on the transformed surface elements should be calculated using the radiation models. Although UV reflective objects will cause scattering, which may result in higher fluence rate values, considering that scattered light requires a more complex algorithm with more environmental information. Therefore, only direct radiation from light sources is considered in the evaluation process.

Since the visibility of the sources from a surface element is independent of the other surface elements, the fluence rate and irradiance calculations for all surface elements can be evaluated simultaneously. This parallel evaluation significantly enhances the computational efficiency. The evaluation process is described in Algorithm 1.
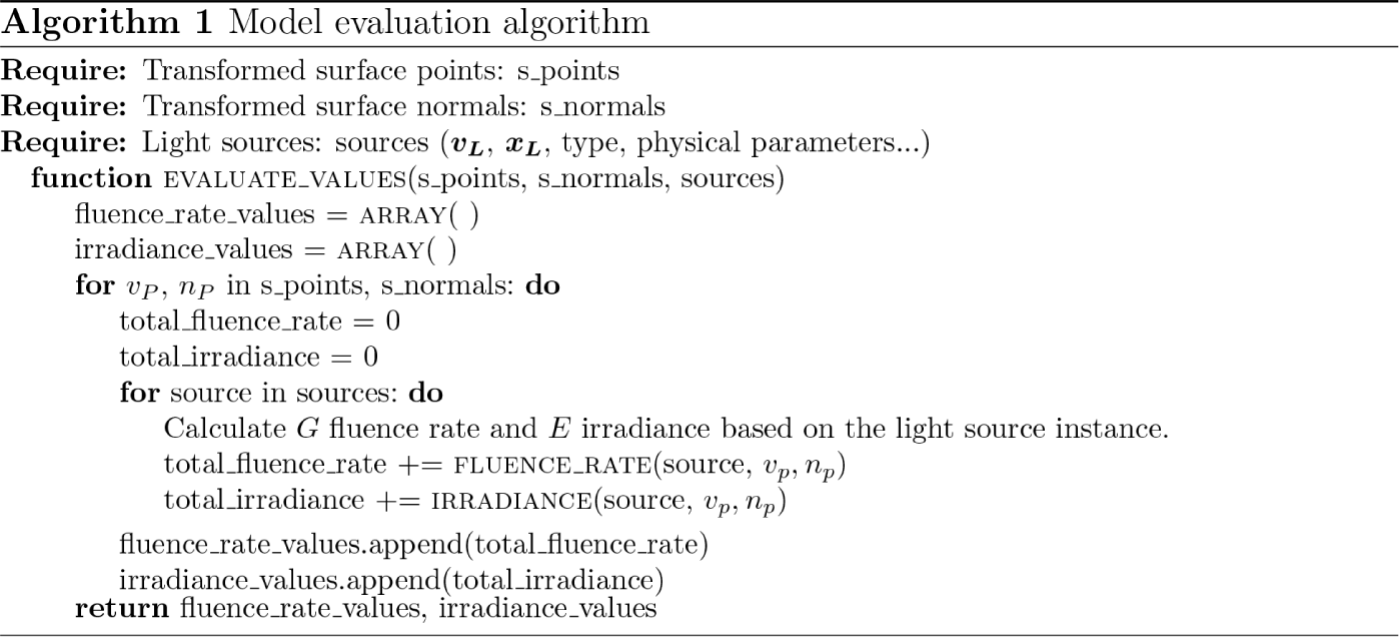


### 2.4 Pathogen Inactivation

After the fluence rate values and received fluence were obtained (by integrating fluence rate values over time), the reduction of pathogens should be determined. For the simulation of the disinfection capability of LPM and LED setups on the example processed object (raw chicken breast), reference studies were selected where pathogen inactivation for similar pathogens was investigated using LPM^30^ and LED sources^31^ on raw chicken breasts. The goal of this comparison is not to directly compare the methods of the two reference studies or to conduct a thorough evaluation of pathogen inactivation on raw chicken breasts, but rather to demonstrate the usability of existing inactivation models in simulations, assuming that these models closely represent the real inactivation process. Using these models in the simulation, the spatial inactivation capability of the process can be estimated.

For a specific object with its optical properties (UV transmittance, reflection, and absorption), as well as surface properties like roughness, pathogen reduction (from UV radiation only) depends on the received fluence, radiation duration (power), pathogen type, and radiation wavelength.^32^ Therefore, microscale shadowing is already accounted for in the inactivation model. Different radiation wavelengths have varying germicidal effects on pathogens. The average germicidal fluence rate from a light source can be estimated from its spectral emission and the pathogen-specific germicidal factor function (GF) (eq 12).^33^
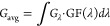
12

where *G*_λ_ (W m^–2^ nm^*–*1^) is the spectral irradiance at wavelength λ. Using this analogy, each light source has an average germicidal power (eq 13), specific to a pathogen, which is a fraction of the total emitted power (*P*_o_).
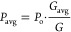
13

In this study, we assume that the power of the light sources and the duration of the processes fall within a range in which the time-dependency of the received fluence can be neglected.

In the first reference study, where pathogen reduction from continuous UV exposure using an LPM source for raw skinless chicken breast was measured,^30^ two-parameter Weibull distribution models were fitted to the observed log reductions for *Salmonella* Enteritidis, *Escherichia coli* EHEC, and *Listeria monocytogenes*, among other bacterial species. A similar experiment using a UV–C LED source to measure the log reduction of *Salmonella* Typhimurium, *Escherichia coli* O157:H7, and *Listeria monocytogenes* was conducted.^31^ To compare the inactivation capabilities of the different light source types on pathogens from the reference studies, Weibull (eq 14) and Chick-Watson models (eq 15) were estimated for each pathogen, as shown in Table 1 along with the estimated average germicidal power ratio, using GF data for the three pathogen types and the emission spectrum of the Nichia NCSU434C LED source and an ideal LPM source (emitting only at 253.7 nm).^32,33^ The average germicidal power ratio (*P*_avg_/*P*_o_) is 1.0 for the LPM source and around 0.65 for the LED source for all of the examined pathogens.
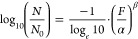
14
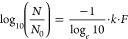
15

**Table 1:** Weibull and Chick-Watson Models of Inactivation for Different Pathogens and Source Types in Raw Chicken Breast^a^

-	-	-	Weibull				Chick-Watson		
Pathogen	Source	*P*_avg_/*P*_o_	α	β	RMSE	*R*^2^	*k*	RMSE	*R*^2^
*Salmonella*	LPM	1.000	0.02	0.14	0.41	0.64	4.82 × 10^–4^	1.04	0.18
-	LED	0.676	290.48	0.58	0.10	0.98	2.39 × 10^–4^	0.23	0.89
*E. coli*	LPM	1.000	0.00	0.09	0.31	0.75	6.51 × 10^–4^	0.90	–0.15
-	LED	0.645	209.59	0.56	0.05	1.00	2.71 × 10^–4^	0.25	0.90
*L. monocyt*.	LPM	1.000	0.00	0.07	0.47	0.41	7.47 × 10^–4^	1.09	–0.17
-	LED	0.640	466.90	0.76	0.05	1.00	2.56 × 10^–4^	0.13	0.97

a Fluence values in the models are given in mJ/cm^2^.

**Figure 7:**
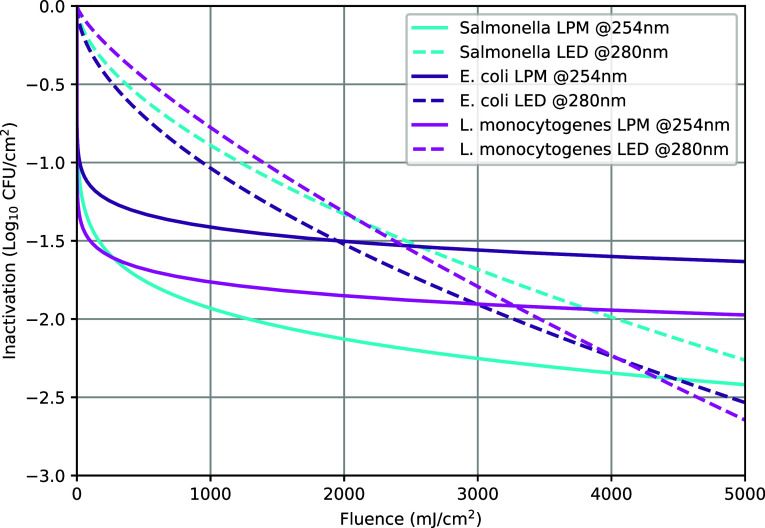
Inactivation curves on raw chicken breasts for different pathogens from the example studies using two different types of light sources.

Since the Chick-Watson model poorly estimates the data from the experiment using the LPM light source, the Weibull models are used in the simulations. Figure 7 displays only the better-fitting Weibull model curves for different pathogen-wavelength combinations in the raw chicken breast.

### 2.5 Received Dose Simulation

The received fluence of a surface element is directly measurable only in the specific case where the fluence rate is equivalent to the irradiance, such as in bench-scale experiments. In other cases, the irradiance or dose is measured. For static experiments, a single irradiance measurement multiplied by the exposure time can be used to determine the received dose. In moving processes, detectors capable of integrating irradiance over time are used to obtain the received doses. The biggest limitation of this method is that it can measure only a single point of the radiation field at a time. To measure doses at multiple locations simultaneously, dosimetry cards can be used for large objects, such as evaluating the internal dose distribution of a UV disinfection cabinet.^34^ To measure dose distribution on smaller objects after a UV irradiation process, radiochromic films (RCF) can be used, as they were to determine dose distribution on the surface of apples in both static and moving experiments.^35,36^ For a detailed dose distribution on the surface of a radiated object, simulations validated by the aforementioned dose-measuring methods can be employed.

**Figure 8:**
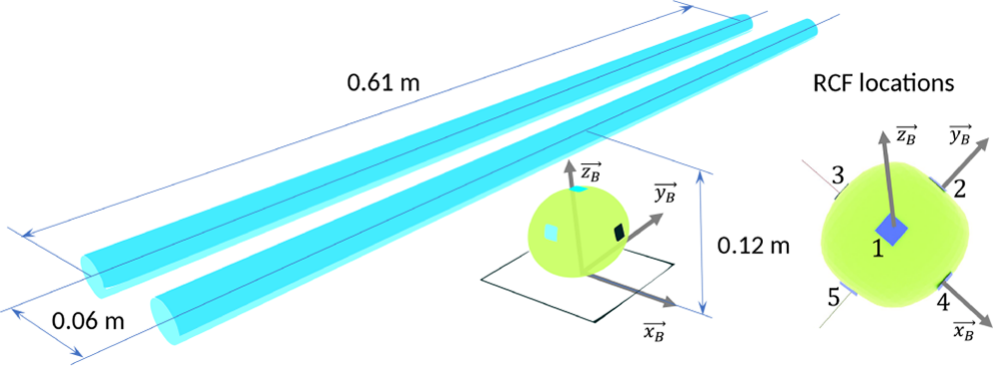
Lamp and apple arrangement in the experiment for dose distribution using RCFs.^36^ Blue rectangles on the apple’s surface are the simulated versions of the RCFs used in the reference study.

To validate our model, dose measurements from a reference study^36^ using RCFs on the surface of an apple were used. In our irradiance simulation, an apple model was rescaled to approximately 6 cm in diameter to match the average apple size, which was used in the reference study. The model was placed at the origin of the XY plane in the base coordinate system (CSB), with its side facing up. By placing virtual RCFs on the surface of an apple model and simulating the conditions of the original experiment, a detailed dose distribution on the apple’s surface and the received fluence distribution can be calculated. Simulating the RCFs, six small planes were placed on the surface of the apple model, where they were placed in the original study: 1—cheek facing up; 6—opposite; 2—stem; 5—opposite (blossom); 3—cheek facing side; 4—opposite (see Figure 8). Five simulations were run in total with slightly changed poses of the virtual RCFs, to simulate apples with different shapes and RCF placement differences.

Our model simulated the lamps in the study as two parallel line light sources at 0.06 m distance, with 0.61 m radiating length, centered above the origin, and with a center axis of [0, 1, 0]. The height of the lamps was set to 0.12 m above the base plane; as in the study, the distance between the lamps and the apples was about 0.06 m.

The emitted optical power was estimated to be 7.5 W for each tube to closely match the irradiance value of the up-facing RCF-1. The apples were radiated in a static position, meaning the irradiance value on the RCFs can be calculated from the measured dose divided by the radiation time (10 s).

### 2.6 Optical Output Power Measurement

Equations 4 and 8 are also applicable to calculate single light sources’ optical power output from light measurements when the perpendicular alignment of the measuring device and the light source is not possible. Or to calculate the output power of complex light shapes that are not precisely measurable, like twin-tube lamps or LED arrays if they consist of LEDs of the same type.

In that particular case, when the surface normal of an irradiance detector is facing the center of a single tube cylindrical lamp and is perpendicular to the center axis of the lamp, if eq 8 is written in the form of expressing radiated optical power (eq 16), it is identical to the Keitz formula,^37,38^ which is used for optical measurements.
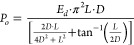
16

**Figure 9:**
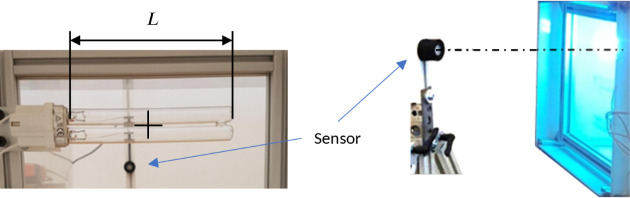
Arrangement of measuring a twin-tube UV–C light source.

A compact, twin-tube UV–C lamp (Philips TUV PL-S 9*W*/2P 1CT) was mounted horizontally to evaluate the diffuse (LSDE) irradiance model for the power measurement. The measurement device was a factory-calibrated Extech SDL-470, using a UV–C (@254 nm) sensor with  accuracy. The sensor was placed to face the geometrical center of the lamp, as seen in Figure 9, and measurements were carried out at multiple distances ranging from 90 to 1200 mm, measured between the face of the detector and the midplane of the lamp. The lamp was modeled as two identical line sources at the centers of the tubes, each with the same emitted optical power. In the virtual environment, the two tubes were placed at [0, 0, 0.007] and [0, 0, −0.007] center positions, with a central axis direction of [0, 1, 0]. The virtual detector was initially placed at the [0.09, 0, 0] position with a surface normal of [−1, 0, 0] and was incrementally moved to the [1.2, 0, 0] position while maintaining its original orientation. The irradiance at the surface of the detector was calculated as the sum of irradiances from the two line sources at each position.

## 3 Results and Discussion

### 3.1 Fluence Distribution

Both in the LPM and LED virtual setups, the fluence rate values for each surface element at each interpolated pose were calculated from the light sources using the corresponding radiation models.

**Figure 10:**
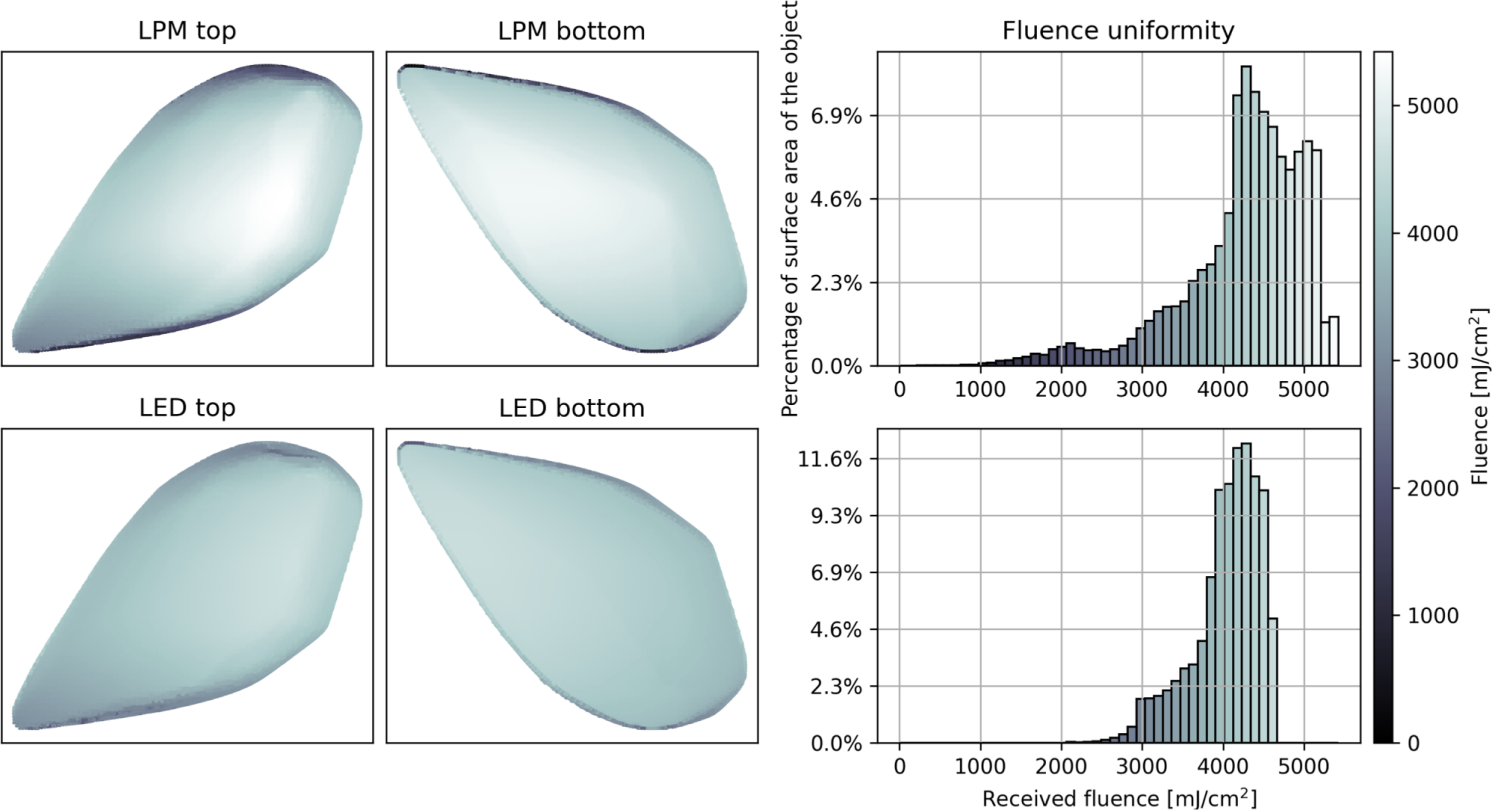
Fluence distribution analysis: Top and bottom view of the radiated object, in the case of the LPM and LED setups (left), histogram of fluence values for fluence uniformity visualization (right).

From the time- (and position-) dependent fluence rate values, the total received fluence was calculated by integrating the fluence rate values over time using the trapezoidal rule. The evenly distributed normalized timestamps were multiplied by the process duration to get the actual fluence for the surface elements. The fluence distribution on the surface of the radiated object is shown in Figure 10.

To examine the uniformity of the received fluence during the process, a histogram shows that the fluence values range between 800 and 5400 mJ/cm^2^, with an average of 4250 mJ/cm^2^ in case of the LPM setup and between 2000 and 4600 mJ/cm^2^ in case of the LED setup, which means that the LED array generates a more uniform fluence distribution with a lower (4030 mJ/cm^2^) average value.

### 3.2 Disinfection Analysis

**Figure 11:**
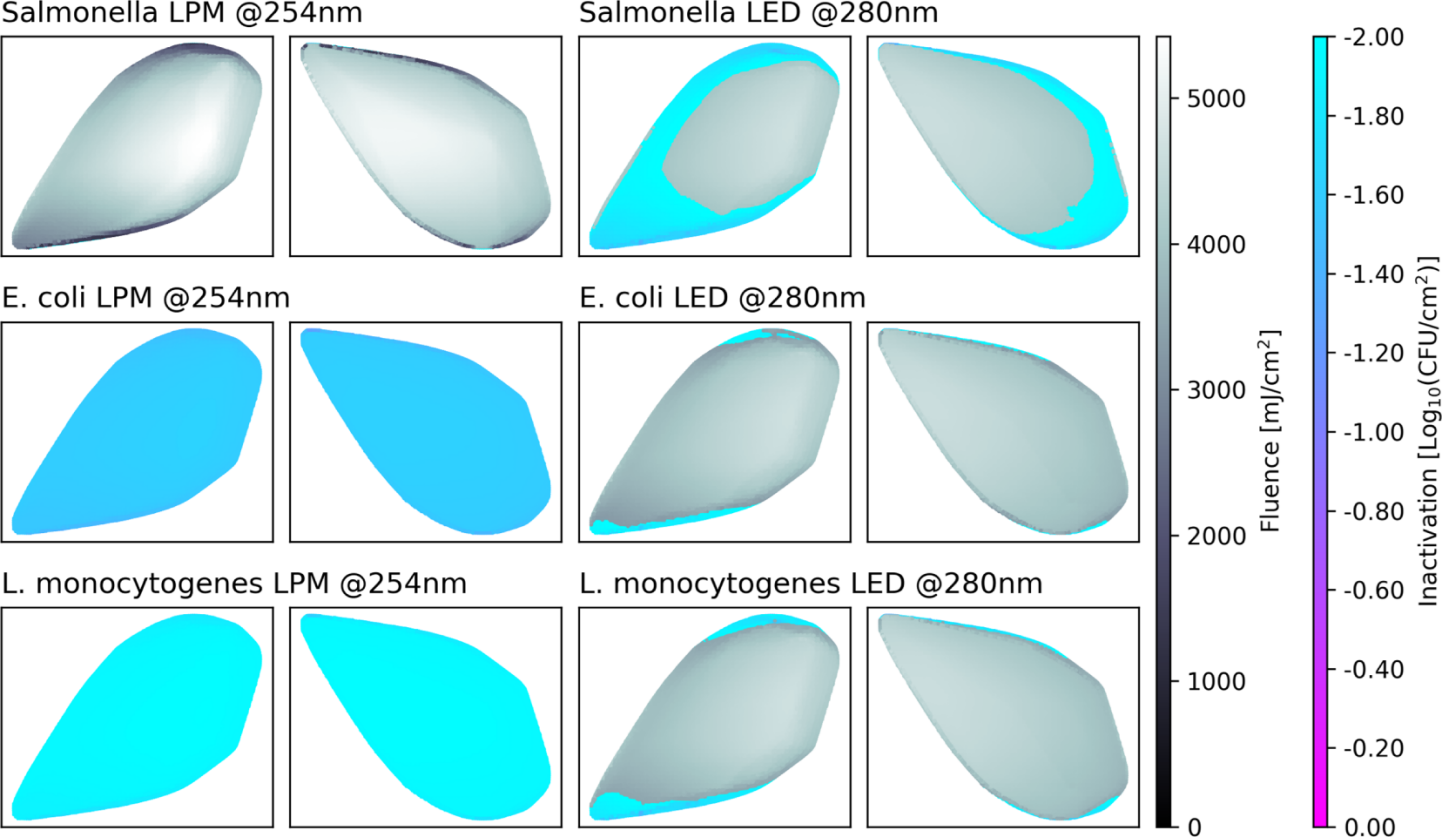
Bacterial inactivation efficiency of the LPM and LED setups for three different types of bacteria.

The received fluence in the LPM setup is at 254 nm, and in the LED case, it is between 260 and 310 nm. Assuming that the wavelength of the NCSU434C LED has a similar effect on the pathogens to what was used in the reference study,^31^ by setting a minimum log inactivation threshold to −2.0 (99%), the reduction effect of the two setups on the three different bacteria can be seen in Figure 11 .

Despite the higher average fluence values and higher average germicidal power ratio (AGPR), the LPM setup achieves the minimum inactivation threshold only for *Salmonella*. In contrast, the LED setup nearly reaches the inactivation threshold for *E. coli* and *L. monocytogenes* in the simulation (except in the least radiated areas) but performs worse against *Salmonella*. For the AGPR calculations, germicidal factors (GFs) of the pathogens were determined under conditions different from those used in the inactivation models. It has been previously observed that pathogen UV sensitivities determined on one type of surface do not always translate well to another.^32^ Therefore, different light source types cannot be directly compared based on the AGPR until GFs are determined using the same surfaces and conditions under which the light sources will be employed for disinfection.

### 3.3 Dose Distribution

**Figure 12:**
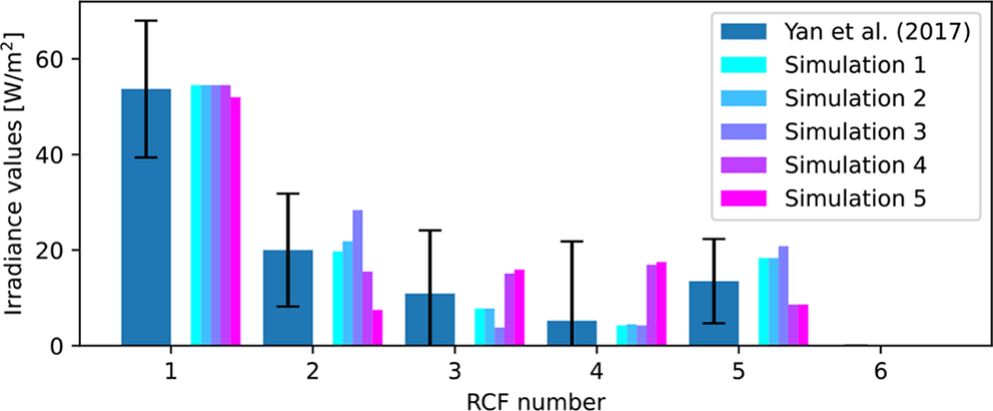
Results of the irradiance simulation compared to the measurements in the reference study.^36^ The error bars show the standard error.

In the reference study, 10 s of UV exposure was applied on the RCFs on apples’ surfaces in a static pose to evaluate dose distribution.^36^ Assuming that the RCF absorbs light at 254 nm completely without transmittance or reflection, the dose value measured with the RCF equals the received irradiance integrated over time. Given the static irradiance field, values of the different RCFs calculated from doses and simulated irradiance values with our model are shown in Figure 12.

**Figure 13:**
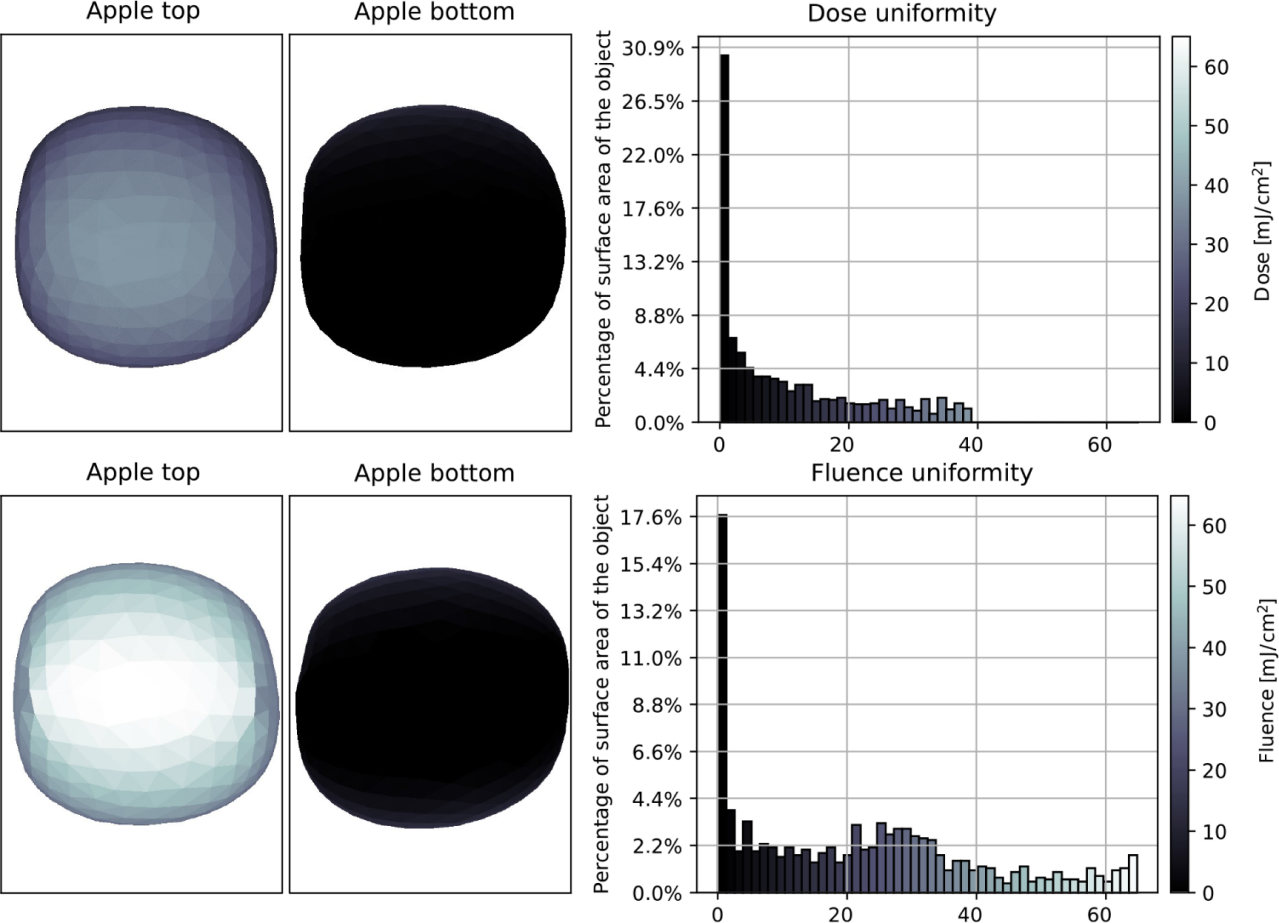
Dose and fluence distribution evaluation on an apple’s surface based on measurements in the reference study.^36^

The simulated virtual RCFs closely match the experimental values, validating that the radiation model of the light sources can accurately calculate the dose and fluence distribution on the surface of the apple (Figure 13).

This means that RCF measurements taken in an operating setup can be translated to a fluence rate distribution on the surface of the radiated object, which can be used to determine the accurate pathogen inactivation capabilities of the UV inactivation process.

### 3.4 LPM Power Measurement

**Figure 14:**
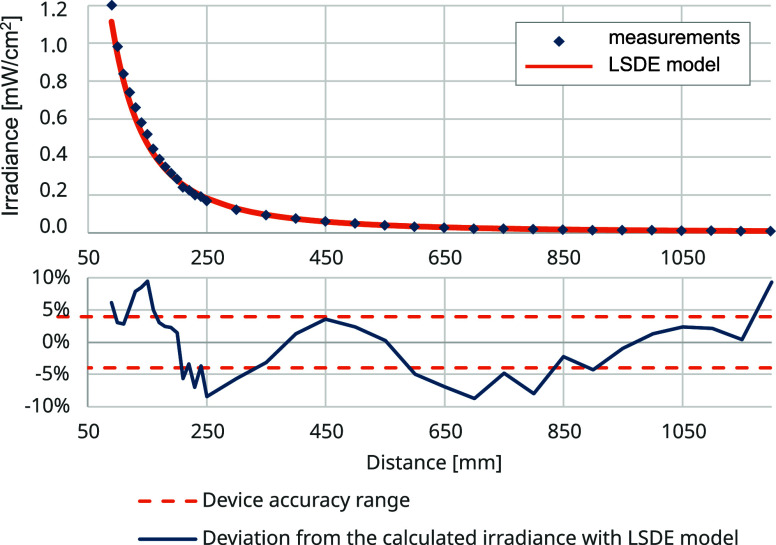
Comparison of the measured irradiance values with the LSDE irradiance model.

The twin-tube LPM light source used in the LPM setup has a nominal optical output power of 2.5 W at 254 nm after 100 h of usage. The lamp was used for no more than 10 h before the measurement. The combined total radiated optical power of the lamp (1.17 W) was calculated as the average power obtained from individual measurements over multiple distances. By substituting the estimated power into the formula for the two line sources, a comparison can be made between the LSDE irradiance model and the measured irradiance values (Figure 14). Relative to the LSDE irradiance model, the irradiance measurements were within a  range along the measurement distances.

### 3.5 Runtime Evaluation

The example 3D object with a convex hull consisting of 43 212 triangles was evaluated by directional-, point-, and diffuse line source light models on a desktop environment with regular multicore CPUs (Intel Core i7-8750H@). The algorithm was constructed in Python using vectorized NumPy^39^ mathematical operations. The average calculation times were:

seconds/point/step/light source for directional light sources,
seconds/point/step/light source for point light sources,
seconds/point/step/light source for line light sources.

This indicates that the model in the LPM setup with 50 intermediate steps (Figure 3) can be evaluated in 3.2 s and the LED setup in 36.7 s. This calculation speed allows the method to be effectively utilized in optimization processes or in soft real-time applications, such as 3D computer-aided design (CAD) environments, where user modifications need to be evaluated quickly.

### 3.6 Model Strengths and Limitations

The developed model offers significant advantages in evaluating the fluence and dose distribution on the surface of objects illuminated by arbitrarily placed heterogeneous light sources. It is effective in dynamic environments, accommodating complex object motion, which makes it highly applicable to real-world scenarios. Additionally, the model enables detailed spatial disinfection analysis by integrating fluence distribution data with pathogen inactivation models, providing a comprehensive assessment of disinfection efficacy. Its use of a generic world coordinate system simplifies the process for design engineers, eliminating the need for specialized knowledge of UV disinfection or radiation modeling and making the tool accessible and practical for a wide range of users.

However, the model has some limitations. It currently does not support all types of UV sources, such as excimer lamps, which could restrict its applicability in certain scenarios. It is also designed to work only with single convex objects and direct radiation, meaning it does not account for scattering, reflection, or macro-scale shadowing effects. Furthermore, the model is not suitable for near-range applications, as the distance between the lamp and the object must be sufficiently large to accurately model LPM light sources as line light sources.

Despite these limitations, the model remains a powerful tool for the fast and accurate evaluation of fluence and dose distributions and for assessing the disinfection capabilities of UV processes.

### 3.7 Discussion

In the design phase of an industrial UV disinfection system, it is crucial to evaluate its effectiveness before finalization. Evaluating different system variants through physical construction is time-consuming and costly compared with computer simulations. Our method aids engineers in designing systems capable of achieving proper disinfection for any object-pathogen-radiation wavelength combination, provided that the pathogen inactivation model for those specific conditions is established. Optimization can be facilitated using algorithms that, given a database of UV sources with their characteristics and system constraints (such as dimensions, complexity, radiation time, and total cost), adjust input parameters to achieve the desired disinfection level.

When a UV system is constructed, detectors can be placed to measure actual irradiance at reference points, enabling the virtual model of the system to act as a digital twin. By evaluating this model, validated by the measured irradiances, variations in the pose and shape of processed objects (detected via computer vision) can be accounted for and process parameters, such as motion speed, can be adjusted in real-time to ensure proper disinfection. The digital twin can also be employed for fault detection: when reference irradiance measurements differ from the model’s corresponding values, it indicates a change in the output power of the light sources.

## 4 Conclusions

This study introduces a unified framework connecting existing radiation models and bench-scale pathogen inactivation models, enabling the UV disinfection process efficiency evaluation. By focusing on an object-based approach, this method supports a general motion model applicable to static and dynamic processes. Through an example, the framework effectively demonstrates differences in fluence distribution and pathogen inactivation between mercury-based cylindrical light sources and LED setups, showcasing its utility in predicting pathogen inactivation for different object-pathogen-radiation wavelength combinations. After validation, it can be utilized for accurate fluence distribution evaluations of 3D objects. The framework’s capability also extends to precise optical power output measurements for complex light geometries.

## Supporting Information Available

Radiation models and calculations, directional light source model, point light source model, and line light source model (PDF)

## Footnotes

a PaShok3D. Chicken breast [3D model]. Sketchfab. Published [21.08.2023]. Available from https://sketchfab.com/3d-models/chicken-breast-5bac86a4617945cd98ead9e1d23ead36. Licensed under CC BY 4.0.
