# Supplementary material for: Fluence and Dose Distribution Modeling of an Ultraviolet Light Disinfection Process for Pathogen Inactivation Efficiency Evaluation
Source: ACS Omega. 2025 Jan 30;10(5):4291–302. doi: 10.1021/acsomega.4c05715 (PMC11822701; doi:10.1021/acsomega.4c05715)
Supplement: Supplementary file 1 — ao4c05715_si_001.pdf [file ao4c05715_si_001.pdf]

SUPPORTING INFORMATION

Fluence and Dose Distribution Modeling of an Ultraviolet Light Disinfection Process for Pathogen Inactivation Efficiency Evaluation

Tamás Dóka* and Péter Horák

Department of Machine and Product Design, Faculty of Mechanical Engineering, Budapest University of Technology and Economics, Műegyetem rkp. 3., H-1111 Budapest, Hungary

E-mail: doka.tamas@gt3.bme.hu

S.1 Radiation models and calculations

To efficiently handle the fluence rate or irradiance contribution calculations of different light sources, their radiation models should be evaluated in the same base framework, to avoid the complex calculations for transforming between the light source reference coordinate system and the object coordinate system. In this framework, a radiated point of an object is described with its position vector ($\vec{v_P}$) and local surface normal ($\vec{n_P}$), and a light source is described with its pose: center point position vector ($\vec{v_L}$) and orientation (local coordinate system ($\boldsymbol{CSL}$)); and its physical properties. The general arrangement of an object point and a light source is shown in Figure S.1.

Figure S. 1. The general arrangement of an object point $\boldsymbol{P}$ with position vector $\vec{\boldsymbol{v_P}}$, local surface normal $\vec{\boldsymbol{n_P}}$ and a line light source described with the position vector of its center point $\vec{\boldsymbol{v_L}}$ and direction vector of its center line $\vec{\boldsymbol{x_L}}$ inside the base coordinate system $\boldsymbol{CSB}$. $\vec{\boldsymbol{p_L}}$ gives the relative position of $\boldsymbol{P}$ from the lamp center point. In the lamp-focused approach, equations are described in the local (lamp) coordinate system $\boldsymbol{CSL}$.

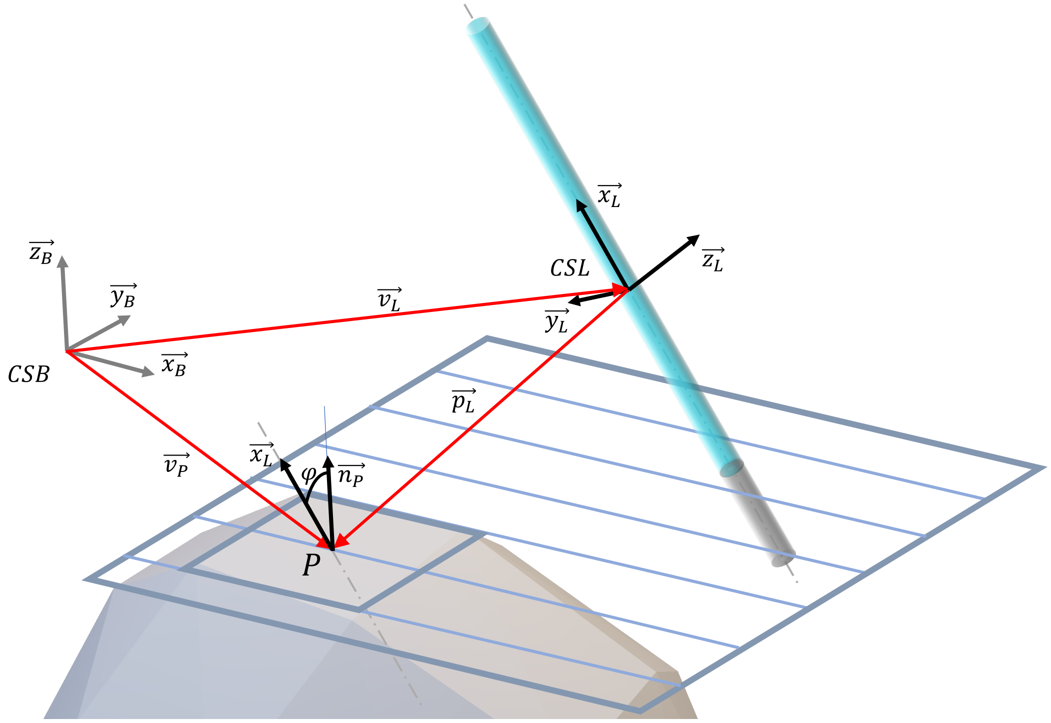

S.1.1 Directional light source model

For directional light, like a collimated light beam, or solar light, the source can be described by its fluence rate $\boldsymbol{F}$ $[W/m^2]$, and direction vector of the light rays $\vec{\boldsymbol{x_L}}$ (Figure S.2).

The fluence rate at the examined point is calculated as written in Equation (1).

$$G\left(\vec{p_L}\right) = H\left(-\vec{x_L} \cdot \vec{n_P}\right) \cdot F \tag{1}$$

And the irradiance can be calculated as shown in Equation (2).

$$E\left(\vec{p_L}\right) = H\left(-\vec{x_L} \cdot \vec{n_P}\right) \cdot F \cdot \left(-\vec{x_L} \cdot \vec{n_P}\right) \tag{2}$$

Where $\boldsymbol{H}$ is the Heaviside step function, which is zero for negative numbers and constant

Figure S. 2. Relations between a directional light source and an arbitrary surface point.

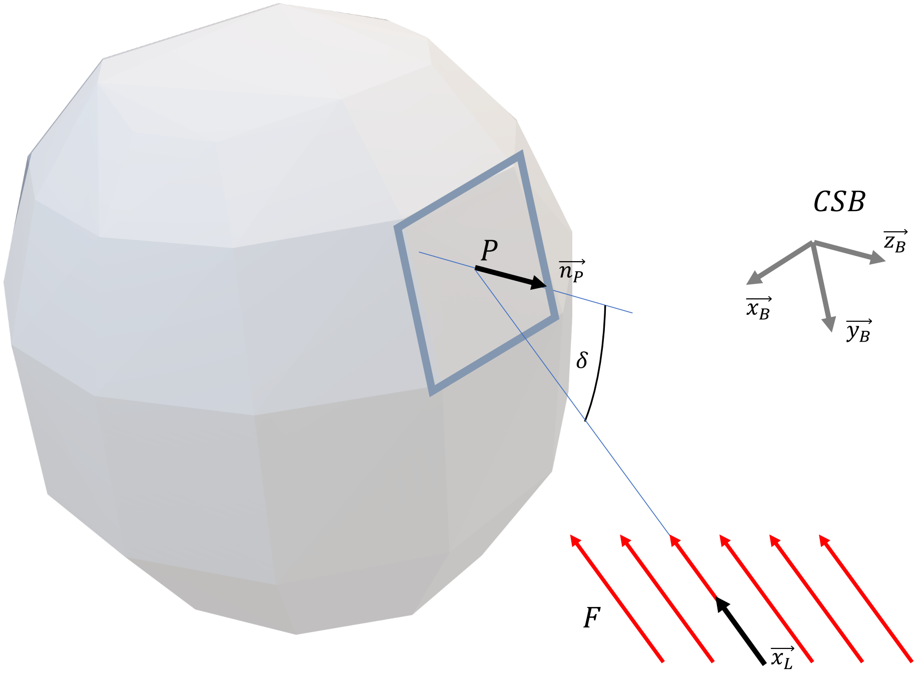

one for non-negative numbers, to consider surface elements that are not facing the light source and, hence, are in shadow.

Algorithmic implementation of the fluence rate and irradiance calculation for directional lights can be seen in Algorithm 1.

Algorithm 1 Directional light source fluence rate and irradiance evaluation algorithm

Require: Surface normal vector n_P
Require: Light direction vector x_L
Require: Light parameter F
  function FLUENCE_RATE_DIRECTIONAL(n_P, x_L, F)
    Calculate $\boldsymbol{G}$ fluence rate as Eq. (1)
    if dot$(-x_L, n_P) \geq 0$ **then return** F
    else return 0
  function IRRADIANCE_DIRECTIONAL(n_P, x_L, F)
    Calculate $\boldsymbol{E}$ irradiance as Eq. (2)
    return FLUENCE_RATE_DIRECTIONAL(n_P, x_L, F) $\cdot$ dot$(-x_L, n_P)$

S.1.2 Point light source model

For point light sources, like most LEDs, the emitter can be described by its total emitted optical power $\boldsymbol{P_o}$, angle-dependent relative intensity function $\boldsymbol{I_{rel}}(\gamma)$, position vector of the

lamp center $\vec{v_L}$ and direction vector of the lamp axis $\vec{x_L}$. Here $\vec{x_L}$ represents the central axis of the point source (Figure S.3).

Figure S. 3. Relations between a point light source and an arbitrary surface point.

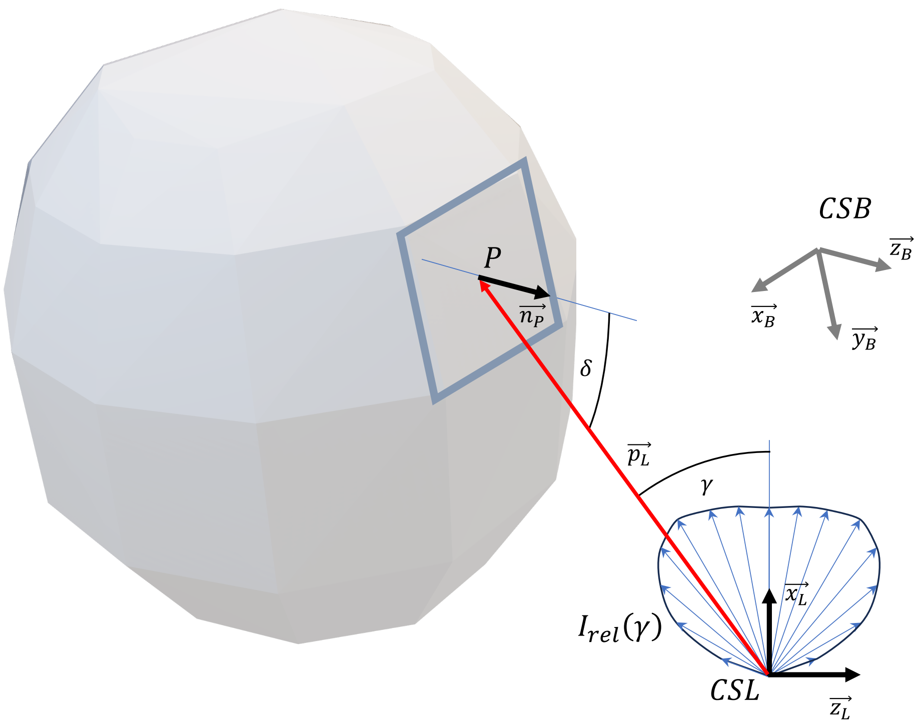

Based on measurements, an LED's real relative intensity function depends both on polar and azimuthal angles[1]. However, measuring the 3D intensity field is challenging, therefore a simplified model is used, assuming that the relative intensity function is axisymmetrical to $\vec{x_L}$. The calculation of fluence rate and irradiance values at a point with position vector $\vec{v_P}$ and with local surface normal $\vec{n_P}$ are similar to directional light sources. For a point source (with position vector of the lamp center $\vec{v_L}$ and direction vector of the lamp central axis $\vec{x_L}$) the connection between the total emitted optical power and the radiated intensity is described in Equation (3).

$$P_o = \int_0^{2\pi} \int_0^{\frac{\pi}{2}} I_{max} \cdot I_{rel}(\gamma) \cdot \sin\gamma \; d\gamma \; d\vartheta \tag{3}$$

where,

- I_{max} $[W/sr]$ is the maximum intensity,
- $I_{rel}(\gamma)$ $[-]$ is the relative intensity function,
- γ [rad] is the emission angle.

Most of the point source light emitters are surface mounted, so the total radiated power is emitted only in a hemisphere. Therefore, the upper limit of the inner integral is $\pi/2$. If the light source is emitting in every direction, the upper limit should be π.

The values of $\boldsymbol{P_o}$ and $\boldsymbol{I_{rel}}(\gamma)$ are usually given by the manufacturer of the LED light source in the product datasheet. Hence, the value of I_{max} can be calculated from Equation (3) either solving the double integral analytically or with a numerical method.

The relative intensity function of the Nichia NCSU434C LED, what is used in the example setup and the estimated directional intensity values can be found in Table 1.

Table 1: Relative and absolute intensity data for a Nichia NCSU434C LED from 0 to 90 degrees. The angle of the light ray is measured from the optical axis.

**Angle from optical axis**	[°]	0	10	20	30	40	50	60	70	80	90
**Relative intensity**	[-]	0.88	0.93	0.98	1.00	0.92	0.70	0.45	0.25	0.05	0.00
**Intensity**	$[W/sr]$	0.032	0.033	0.035	0.036	0.033	0.025	0.016	0.009	0.002	0.000

After calculating I_{max}, the fluence rate at the examined point can be described with Equation (4).

$$G(\vec{p_L}) = H\left(-\vec{p_L} \cdot \vec{n_P}\right) \frac{I_{max} \cdot I_{rel}\left(\cos^{-1}\left(\frac{\vec{x_L} \cdot \vec{p_L}}{\|\vec{p_L}\|}\right)\right)}{\|\vec{p_L}\|^2} \tag{4}$$

Also, the irradiance received from the point source can be calculated similarly by multiplying the fluence rate value with the cosine of the incident angle (δ [rad]), which can be expressed with $\boldsymbol{\vec{p_L}}$ and $\boldsymbol{\vec{n_P}}$ (Equation (5)), which is the same equation used and validated previously[2], except the Heaviside function, which makes the equation generally usable.

$$E(\vec{p_L}) = H\left(-\vec{p_L} \cdot \vec{n_P}\right) \frac{I_{max} \cdot I_{rel}\left(\cos^{-1}\left(\frac{\vec{x_L} \cdot \vec{p_L}}{\|\vec{p_L}\|}\right)\right) \cdot \left(-\vec{p_L} \cdot \vec{n_P}\right)}{\|\vec{p_L}\|^3} \tag{5}$$

The algorithmic representation of the point source fluence rate and irradiance calculations is shown in Algorithm (2).

Algorithm 2 Point light source fluence rate and irradiance evaluation algorithm

Require: Point position vector: v_P
Require: Surface normal vector: n_P
Require: Light position vector: v_L
Require: Light direction vector: x_L
Require: Light parameter: I_{max}
Require: Relative intensity function: REL_INTENSITY(angle)

```
function FLUENCE_RATE_POINT(v_P, n_P, v_L, x_L, I_max)
    Calculate point position from light center
    p_L = v_P - v_L
    Calculate cosine of light incidence angle (c_d) and cosine of emission angle (c_g)
    c_d = dot(-p_L, n_P) / norm(p_L)
    c_g = dot(p_L, x_L) / norm(p_L)
    Calculate G fluence rate as Eq. (4)
    if c_d ≥ 0 then return I_max · REL_INTENSITY(arccos(c_g)) / norm(p_L)^2
    else return 0
function IRRADIANCE_POINT(v_P, n_P, v_L, x_L, I_max)
    Calculate point position from light center
    p_L = v_P - v_L
    Calculate cosine of light incidence angle (c_d)
    c_d = dot(-p_L, n_P) / norm(p_L)
    Calculate E irradiance as Eq. (5)
     return FLUENCE_RATE_POINT(v_P, n_P, v_L, x_L, I_max) ·c_d
```

S.1.3 Line light source model

Cylindrical light sources can be modeled as line, surface, or volumetric sources. Among these, the line source models are the simplest yet still adequate with considerably low inaccuracies when the distance between the radiated point and the source is relatively high compared to the radius of the lamp[3–5]. A lamp can be described by its total emitted optical power $\boldsymbol{P_o}$, radiating length $\boldsymbol{L}$, position vector of the lamp center $\vec{\boldsymbol{v_L}}$ and direction vector of the lamp axis $\vec{\boldsymbol{x_L}}$. The relations for calculation of fluence rate and irradiance values at a point with position vector $\vec{\boldsymbol{v_P}}$ and with local surface normal $\vec{\boldsymbol{n_P}}$ can be seen in Figure S.1, where the vector pointing from the center of the lamp to the examined point is $\vec{\boldsymbol{p_L}}$. All of the position and direction vectors are represented in the base coordinate system ($\boldsymbol{CSB}$).

For fluence rate calculations, where the incident angle on the surface is not considered,

the models are well-known for specular sources like neon lamps (LSSE - line source spherical emission model) and for diffuse light sources like fluorescent and LPM UV-C emitters (LSDE - line source diffuse emission model)[6]. In these models, it is assumed that the examined point in the radiated field is radiated by the total length of the light source ($\boldsymbol{L}$). However, in a general arrangement, when the local surface plane of the examined point intersects the lamp at its radiating length, radiation is received only from the part of the lamp that extends above the examined point. These models are calculated in a reference coordinate system $\boldsymbol{CSR}$, which is aligned with $\boldsymbol{CSL}$ and shifted from the light center with $\vec{\boldsymbol{p_L}} \cdot \vec{\boldsymbol{x_L}}$ along $\vec{\boldsymbol{x_L}}$. Therefore, the fluence rate value at a given point should be calculated as described in Equation (6) for specular and in Equation (7) for diffuse light sources.

$$G_s(\vec{\boldsymbol{p_L}}) = \frac{P}{4\pi L} \int_{L_-}^{L_+} \frac{1}{(D^2 + x^2)} dx \tag{6}$$

$$G_d(\vec{\boldsymbol{p_L}}) = \frac{P}{\pi^2 L} \int_{L_-}^{L_+} \frac{D}{\sqrt{(D^2 + x^2)^3}} dx \tag{7}$$

Where,

- $D[m]$ is the distance between the examined point and the centerline of the lamp.
- $L_+[m]$ is the distance of the end of the effective radiating part of the light source along the $\vec{\boldsymbol{x_L}}$ direction in $\boldsymbol{CSR}$.
- $L_-[m]$ is the distance of the end of the effective radiating part of the light source opposite the $\vec{\boldsymbol{x_L}}$ direction in $\boldsymbol{CSR}$.

The value of $\boldsymbol{D}$ can be calculated as shown in Equation (8).

$$D = ||\vec{p_L} - (\vec{x_L} \cdot \vec{p_L}) \cdot \vec{x_L}|| \tag{8}$$

The relation between the light source and the examined point in a lamp-focused perspective can be seen in Figure S.4.

Figure S. 4. Relation between the line light source and the radiated point in a lamp-focused perspective.

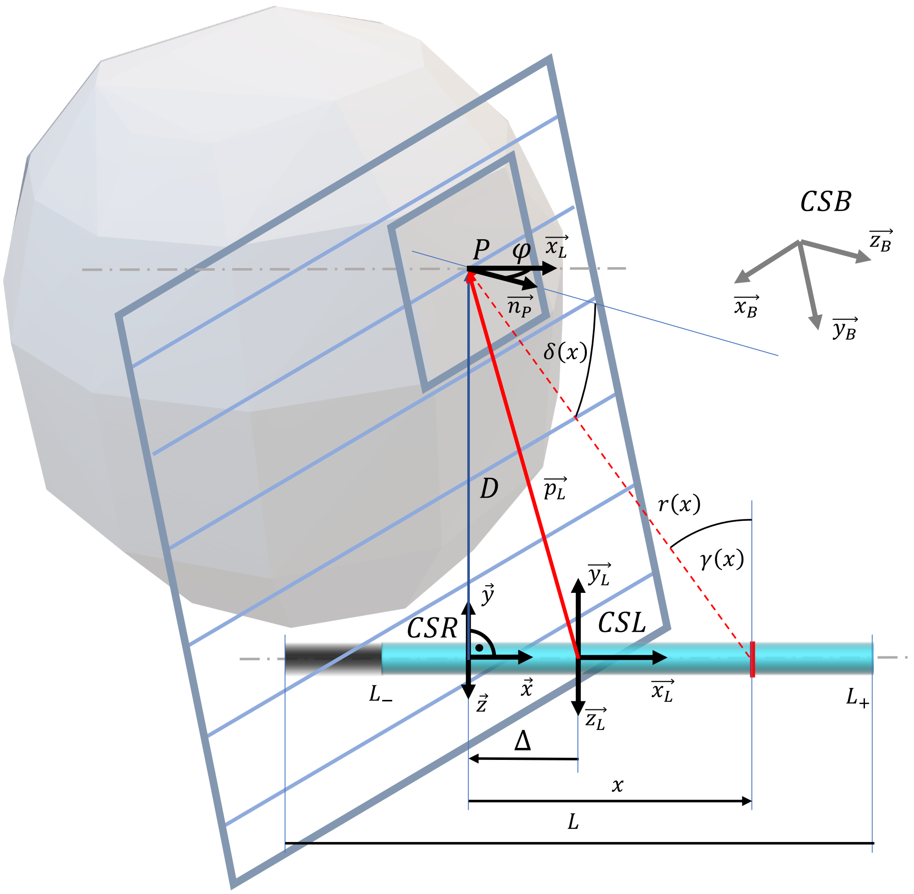

Originally the integral limits ($\boldsymbol{L_+}$, $\boldsymbol{L_-}$) when the whole lamp radiates the point are calculated as shown in Equations (9 - 10).

$$L_+ = \frac{L}{2} - \vec{x_L} \cdot \vec{p_L} \tag{9}$$

$$L_- = -\frac{L}{2} - \vec{x_L} \cdot \vec{p_L} \tag{10}$$

The relation between the light source and the point should be considered to calculate the ends of the *effective* radiating length of the light source for a given point. For this, it should be determined whether the intersection of the local surface plane of the point and

the light source's centerline happens within the lamp's radiating length or outside of it. The intersection is determined by the parameter ***t***, which shows the ratio of the signed distance of the intersection from the light center to the distance of the end of the lamp from the lamp center (L/2). The parameter ***t*** is calculated as shown in Equation (11).

$$t = \frac{\vec{n_p} \cdot \vec{p_L}}{\frac{L}{2} \cdot (\vec{n_p} \cdot \vec{x_L})} \tag{11}$$

If the absolute value of parameter ***t*** is smaller than one, it means that the intersection happens within the lamp (see Figure S.5), hence the corresponding integral limit should be modified based on the value of ***t***, described in Equation (12):

$$\begin{cases} \vec{n_p} \cdot \vec{x_L} < 0, \text{then } L_+ = \min(\max(t; -1); 1) \cdot \frac{L}{2} - \vec{x_L} \cdot \vec{p_L} \\ \vec{n_p} \cdot \vec{x_L} \geq 0, \text{then } L_- = \min(\max(t; -1); 1) \cdot \frac{L}{2} - \vec{x_L} \cdot \vec{p_L} \end{cases} \tag{12}$$

Figure S. 5. Visual representation of the effective radiating segment of the light source for the examined point ***P***.

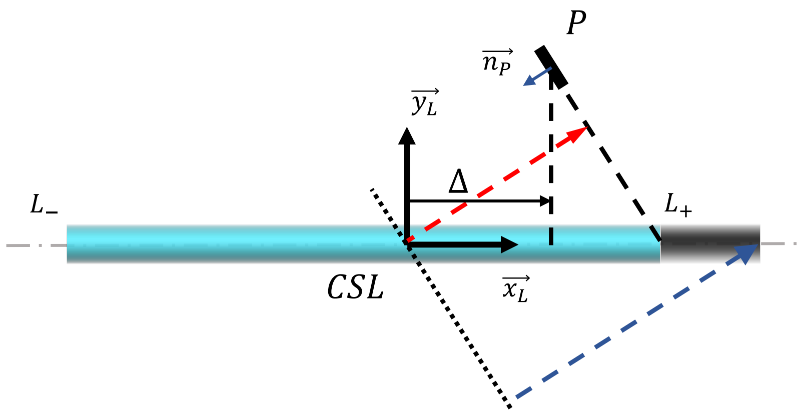

Knowing the integral limits, the fluence rate values can be obtained in a closed form both for specular (Equation (13)) and diffuse cases (Equation (14)). These equations are equivalent to those summarized earlier[7,8], except for having the integral limits in the general form.

$$G_s(\vec{p_L}) = \frac{P_o}{4\pi LD} \cdot \left[tan^{-1}\left(\frac{L_+}{D}\right) - tan^{-1}\left(\frac{L_-}{D}\right)\right] \tag{13}$$

$$G_d(\vec{p_L}) = \frac{P_o}{\pi^2 LD} \cdot \left[\frac{L_+}{\sqrt{D^2 + {L_+}^2}} - \frac{L_-}{\sqrt{D^2 + {L_-}^2}}\right] \tag{14}$$

For irradiance calculation, the incident angle of the radiation beam on the local surface from every differentially small element of the light source is considered. The problem of using fluence rate values instead of irradiance when the reactor wall's surface normal is considered during computational fluid dynamics (CFD) simulations of ultraviolet photoreactors was addressed. The proposed method calculates fluence rate components in radial and axial directions separately, and then the total irradiance from the lamp is derived from these components and the surface normal[9].

However, the received irradiance from a differentially small part of the lamp can be directly calculated with the following equation for specular light sources (Equation (15)),

$$dE_s(\vec{p_L}) = \frac{P_o}{4\pi L} \cdot \frac{\cos\delta(x)}{r^2(x)} \tag{15}$$

and for diffuse light sources (Equation (16)).

$$dE_d(\vec{p_L}) = \frac{P_o}{\pi^2 L} \cdot \frac{\cos\gamma(x) \cdot \cos\delta(x)}{r^2(x)} \tag{16}$$

Where,

- γ [rad] is the angle of emission from the differentially small emitter element.
- δ [rad] is the incident angle to the surface normal.
- r [m] is the distance between the radiated point and the small emitter element.

Using these, the total received irradiance from the effective radiating part of the lamp in the specular case can be calculated as shown in Equation (17):

$$E_s(\vec{p_L}) = \frac{P_o}{4\pi L} \int_{L_-}^{L_+} \frac{(C_1 \cdot x + C_2)}{\sqrt{(D^2+x^2)^3}}, dx \tag{17}$$

Which yields (Equation (18)):

$$E_s(\vec{p_L}) = \frac{P_o}{4\pi L} \left[\left(\frac{C_2 \cdot L_+ - C_1 \cdot D^2}{D^2\sqrt{D^2 + {L_+}^2}} \right) - \left(\frac{C_2 \cdot L_- - C_1 \cdot D^2}{D^2\sqrt{D^2 + {L_-}^2}} \right) \right] \tag{18}$$

In case of a diffuse light source (Equation (19)):

$$E_d(\vec{p_L}) = \frac{P_o}{\pi^2 L} \int_{L_-}^{L_+} \frac{D\,(C_1 \cdot x + C_2)}{(D^2+x^2)}, dx \tag{19}$$

Which yields (Equation (20)):

$$E_d(\vec{p_L}) = \frac{P_o}{\pi^2 L} \cdot \frac{1}{2D^2} \left[\frac{D\,(C_2 \cdot L_+ - C_1 \cdot D^2)}{D^2 + {L_+}^2} - \frac{D\,(C_2 \cdot L_- - C_1 \cdot D^2)}{D^2 + {L_-}^2} + C_2 \left(\tan^{-1}\left(\frac{L_+}{D}\right) - \tan^{-1}\left(\frac{L_-}{D}\right) \right) \right] \tag{20}$$

For a given light-object point relation, the constants $\boldsymbol{C_1}$, which is the cosine of the angle between the surface normal ($\vec{\boldsymbol{n_p}}$) and the direction vector of the centerline of the light source ($\vec{\boldsymbol{x_L}}$), and $\boldsymbol{C_2}$ which is the height of the origin of $\boldsymbol{CSR}$ above the local surface plane in Equations (17-20), can be calculated for each examined point as seen in Equations (21-22).

$$C_1 = \vec{x_L} \cdot \vec{n_P} \tag{21}$$

$$C_2 = (-\vec{p_L} \cdot \vec{n_P}) + (\vec{x_L} \cdot \vec{p_L}) \cdot C_1 \tag{22}$$

The fluence rate and irradiance calculations for cylindrical light sources is written in Algorithm 3.

Algorithm 3 Line light source fluence rate and irradiance evaluation algorithm

Require: Point position vector: v_P
Require: Surface normal vector: n_P
Require: Light position vector: v_L
Require: Light direction vector: x_L
Require: Light parameters: P_o, L

```
function CALCULATE_VALUES(v_P, n_P, v_L, x_L, P_o, L)
    Calculate point position vector from the light center
    p_L = v_P − v_L
    Calculate shift from CSL to CSR
    shift = dot(x_L, p_L)
    Calculate lamp center height from plane
    height = dot(−p_L, n_P)
    Calculate arrangement constants
    C_1 = dot(x_L, n_P)
    C_2 = height + shift · C_1
    Calculate initial integral limits
    L_+ = L/2 − shift
    L_− = −L/2 − shift
    Calculate intersection parameter
    t = −height/(L/2 · C_1)
    Update integral limits
    if C_1 < 0 then
        L_+ = min(max(t, −1), 1) · L/2 − shift
    else
        L_− = min(max(t, −1), 1) · L/2 − shift
    Calculate point-centerline distance:
    D = norm(p_L − shift · x_L)
    return P_o, L, D, C_1, C_2, L_+, L_−
function FLUENCE_RATE_LINE(v_P, n_P, v_L, x_L, P_o, L)
    values = CALCULATE_VALUES(v_P, n_P, v_L, x_L, P_o, L)
    Calculate and return G fluence rate as Eq. (14)
    return = G(values)
function IRRADIACE_LINE(v_P, n_P, v_L, x_L, P_o, L)
    values = CALCULATE_VALUES(v_P, n_P, v_L, x_L, P_o, L)
    Calculate and return E irradiance as Eq. (20)
    return = E(values)
```

References

(1) Kheyrandish, A.; Taghipour, F.; Mohseni, M. UV-LED radiation modeling and its applications in UV dose determination for water treatment. *J. Photochem. Photobiol. A Chem.* **2018**, *352*, 113–121.

(2) Lai, P.-Y.; Liu, H.; Ng, R. J. H.; Wint Hnin Thet, B.; Chu, H.-S.; Teo, J. W. R.; Ong, Q.;

Liu, Y.; Png, C. E. Investigation of SARS-CoV-2 inactivation using UV-C LEDs in public environments via ray-tracing simulation. *Scientific Reports* **2021**, *11*.

(3) Quan, Y.; Pehkonen, S. O.; Ray, M. B. Evaluation of three different lamp emission models using novel application of potassium ferrioxalate actinometry. *Ind. Eng. Chem. Res.* **2004**, *43*, 948–955.

(4) Pareek, V.; Chong, S.; Tadé, M.; Adesina, A. A. Light intensity distribution in heterogenous photocatalytic reactors. *Asia-Pacific Journal of Chemical Engineering* **2008**, *3*, 171–201.

(5) Boyjoo, Y.; Ang, M.; Pareek, V. Lamp emission and quartz sleeve modelling in slurry photocatalytic reactors. *Chem. Eng. Sci.* **2014**, *111*, 34–40.

(6) Akehata, T.; Shirai, T. Effect of light-source characteristics on the performance of circular annular photochemical reactor. *J. Chem. Eng. Japan* **1972**, *5*, 385–391.

(7) Duran, J. E.; Taghipour, F.; Mohseni, M. Irradiance modeling in annular photoreactors using the finite-volume method. *J. Photochem. Photobiol. A Chem.* **2010**, *215*, 81–89.

(8) Deng, B.; Peng, S.; Ye, K.; Zhao, B. Axisymmetric radiation intensity model for annular reactors. *AIChE J.* **2021**, *67*.

(9) Chen, W.; Sun, Y.; Hu, M.; Deng, B. Radiation modeling and the simulation of the microorganism disinfection in ultraviolet reactors. *Ind. Eng. Chem. Res.* **2023**,
